# Breast Cancer Screening Among Women With Intellectual Disability in Denmark

**DOI:** 10.1001/jamanetworkopen.2022.48980

**Published:** 2023-01-03

**Authors:** Trine Allerslev Horsbøl, Susan Ishøy Michelsen, Tina Harmer Lassen, Knud Juel, Janne Bigaard, Christina Engel Hoei-Hansen, Ilse Vejborg, Lau Caspar Thygesen

**Affiliations:** 1 National Institute of Public Health, University of Southern Denmark, Copenhagen, Denmark; 2 Prevention and Information, Danish Cancer Society, Copenhagen, Denmark; 3 Department of Paediatrics and Adolescent Medicine, Copenhagen University Hospital–Rigshospitalet, Copenhagen, Denmark; 4 Department of Clinical Medicine, University of Copenhagen, Copenhagen, Denmark; 5 Department of Breast Examinations, Copenhagen University Hospital Herlev Gentofte, Copenhagen, Denmark

## Abstract

**Importance:**

Breast cancer–specific mortality is increased among women with intellectual disability (ID), and knowledge about participation in breast cancer screening in this group is needed.

**Objective:**

To examine participation in the Danish national breast cancer screening program among women with ID compared with women without ID.

**Design, Setting, and Participants:**

This dynamic population-based cohort study assessed participation in the Danish national breast cancer screening program initiated in 2007, targeting women aged 50 to 69 years with a screening interval of 2 years. In all, 6357 women with ID born between 1941 and 1967 and eligible for the screening program were identified in national registers. Women entered the study between January 1, 2007, and December 31, 2017. Subsequently, 273 women were excluded due to a history of carcinoma in situ or breast cancer, and 489 due to registration errors in registers. Each woman was individually age-matched with 10 women without ID (reference group). All women were followed up until March 31, 2021, or censoring (due to death, carcinoma in situ, or breast cancer). Data were analyzed from December 1, 2021, to June 31, 2022.

**Exposures:**

Intellectual disability was defined as being registered with an ID diagnosis or a diagnosis most likely leading to ID or residing at an institution for persons with ID.

**Main Outcomes and Measures:**

Participation in breast cancer screening (fully, partly, and never).

**Results:**

A total of 5595 women with ID and 49 423 age-matched women in the reference group were included in the analysis. Of these, 2747 women with ID (49%) and 24 723 in the reference group (50%) were 50 years of age at study entry; for those older than 50 years, the median age was 51 years (IQR, 50-58 years) in both groups. In all, 1425 women with ID (25%) were fully screened according to guidelines for the Danish breast cancer screening program compared with 30 480 women in the reference group (62%). Women with ID had nearly 5 times higher odds of never being screened compared with the reference group (odds ratio, 4.90 [95% CI, 4.60-5.22]). In all, 2498 women with ID (45%) and 6573 in the reference group (13%) were never screened. The proportion of never-screened women increased with severity of ID, from 834 of 2287 (36%) among women with mild ID to 173 of 212 (82%) among women with profound ID.

**Conclusions and Relevance:**

The findings of this cohort study suggest that women with ID are markedly less likely to participate in breast cancer screening compared with women without ID. These findings further suggest a need for tailored guidelines and approaches for breast cancer screening in this group of women.

## Key Points

**Question:**

Do women with intellectual disability (ID) participate in the Danish national breast cancer screening program to the same degree as other women at their age?

**Findings:**

In this nationwide cohort study of 5595 Danish women with ID, markedly lower participation in breast cancer screening was found compared with women without ID; 45% of women with ID and 13% of women without ID were never screened. Screening participation decreased with increasing severity of ID.

**Meaning:**

These findings suggest that a considerable proportion of Danish women with ID are not screened according to guidelines that exist to detect breast cancer at early stages.

## Teaser

This cohort study examines participation in the Danish national breast cancer screening program among women with intellectual disability compared with women without intellectual disability.

## Introduction

Recently published studies have found markedly increased overall^1,2^ and breast cancer–specific^1^ mortality among women with intellectual disability (ID). Mammography screening has proven efficient to detect breast cancer at early stages. This enables early initiation of treatment, which contributes to reduced late effects and increased survival.^3,4,5,6,7^

Previous studies^8,9,10,11,12,13,14,15^ have shown lower participation in breast cancer screening among women with ID compared with women without. However, the proportions of women with and without ID who participate in breast cancer screening differ widely across studies, settings, and countries (4%-64%).^8,9,10,11,12,13,14,15^ Breast cancer screening participation among women with ID has not been investigated in Denmark. The Danish health care system is tax-financed, and all citizens have universal access to free health care services, including cancer screening.^16^ Thus, it is an ideal setting to evaluate participation independently of barriers caused by income and access to health care insurance.

Attendance for the Danish national breast cancer screening program is high, and in the last screening round, 84% of invited women participated.^17^ Despite universal access to the screening program, women with chronic diseases, multimorbidity, or mental illness are less likely to participate.^18,19^ Further, nonparticipation has also been found to be associated with low social status, no access to a vehicle, and being of non-Danish origin.^20^

Life expectancies are markedly lower among individuals with ID than the general population (L.C.T., T.H.L., T.A.H., et al; unpublished data; June 2022).^21^ Further, all aspects of care provision, planning, and coordination have been found to deteriorate in this group of potentially vulnerable individuals.^21^ The primary aim of this study was to examine participation in the Danish national breast cancer screening program among women with ID compared with women without ID.

## Methods

We conducted a dynamic population-based cohort study among women with ID and age-matched women in a reference group. All Danish residents are given a unique personal identification number, which was used to combine data from several high-quality Danish national registers.^22,23^ According to Danish law, ethical review and informed consent from participants are not required in register-based studies. In agreement with the General Data Protection Regulation, the present study is registered at University of Southern Denmark, Copenhagen. The study followed the Strengthening the Reporting of Observational Studies in Epidemiology (STROBE) reporting guideline.

### Context

The Danish national breast cancer screening program was initiated in 2007 and fully implemented at the end of 2010. The target group of the program consists of women aged 50 to 69 years, and the screening interval is scheduled every 2 years. The program is organized in screening rounds, each lasting approximately 2 years. During each screening round, all women in the target group are invited to breast cancer screening. Until now, 6 national screening rounds have been completed, the last one ending March 31, 2021. Women are invited electronically unless they actively unregister from the program, which can be done electronically or by contacting the local secretariates for breast cancer screening. Instructions on how to unregister are provided in the invitation.

Before 2007, there were local screening programs in some parts of Denmark. Thus, some of the women who were older than 50 years during the first national screening round may have been screened previously.^24,25^

### Study Population

Women born between 1941 and 1967 were eligible for inclusion. Those born between 1941 and 1956 entered the study at 50 to 69 years of age on January 1, 2007. Those born between 1957 and 1967 entered the study when they turned 50 years of age between January 1, 2007, and December 31, 2017.

We identified a cohort of women with ID from different sources, as no systematic registration of ID exists in Denmark. A detailed description of establishment of the cohort is described elsewhere (L.C.T., T.H.L., T.A.H., et al; unpublished data; June 2022). Briefly, most women were identified in the Danish National Patient Register that includes data on all inpatient (since 1977) and outpatient (since 1995) hospital contacts,^26^ and the Danish Psychiatric Central Research Register, which holds information on all contacts to psychiatric departments since 1969.^27^ They were registered with an ID diagnosis (mild, moderate, severe, profound, or unknown severity) or a diagnosis most likely leading to ID (Down syndrome, selected congenital metabolic disorders, congenital malformations, and chromosomal disorders) (eTable 1 in Supplement 1). Further, women with ID due to cerebral palsy were identified in the Danish Cerebral Palsy Registry, which contains information on persons with cerebral palsy born from 1950 and onwards in the Eastern part of Denmark.^28^ Last, we identified women with permanent residence at institutions for persons with ID.^29^ Consequently, the cohort consisted of 5 diagnostic groups most likely leading to ID: ID diagnosis, cerebral palsy with ID, Down syndrome, metabolic disorder, and congenital malformation and/or chromosomal disorder. A sixth group was identified through institutions (unknown diagnosis). The same women could appear in several of these groups, as they could be recorded with more than 1 diagnosis or identified both through an institution and 1 or more of the inclusion diagnoses.

In all, 6357 women with ID were identified and matched individually on date of birth to 10 women without any of the inclusion diagnoses for ID. Subsequently, we excluded women with a history of carcinoma in situ or breast cancer or with registration errors (eFigure 1 in Supplement 1).

### Participation in Breast Cancer Screening

Information on breast cancer screening was obtained from the Danish Quality Database for Mammography Screening, containing information on invitations, participation, and results of screening. The database was established in 2007 and is based on data from the 5 different booking systems, the Danish National Patient Register, and the Danish National Pathology Registry.^30^ Information on all women in the study was available until March 31, 2021, when the sixth national screening round was completed.

We generated an overall measure of screening participation, categorized as fully screened, partly screened, and never screened. For all birth year cohorts of women, we estimated how many of the 6 invitation rounds in the screening program they were eligible for. We downgraded this number with 1 invitation round for all women to allow for administrative delay and for variation in the exact start and ending dates across geographical regions. Women who died or were diagnosed with carcinoma in situ or breast cancer were censored at that date, and the number of potential screening rounds was adjusted accordingly. Thus, the number of potential screening rounds was allocated individually.

Fully screened women were invited to at least their allocated number of potential screening rounds and participated in all of them. Partly screened women were invited to fewer screening rounds than they potentially should have been or participated in fewer rounds than they were invited to. Never screened women were not invited to any screening rounds or did not participate in any of the rounds they were invited to.

### Covariates

Using the National Patient Register,^26^ we obtained information on diagnoses registered in a period of 10 years prior to study entry to calculate the Charlson Comorbidity Index of physical comorbidity.^31,32,33^ We categorized Charlson Comorbidity Index scores as 0, 1 to 2, and 3 or greater (higher scores indicate increased severity of comorbid conditions). From this register,^26^ we also obtained information on psychiatric comorbidity, defined as any contact with a psychiatric hospital department for psychiatric diagnoses other than ID within the last 10 years.

Using registers held by Statistics Denmark,^34^ we obtained information on the highest attained educational level, work status, and cohabitation status the year prior to study entry. Additionally, information on country of origin (Danish, immigrant or descendant from a Western country, or immigrant or descendant from a non-Western country) was obtained from the Danish Civil Registration System. This information was assessed in the study because we considered it a possible confounder.^29^

### Statistical Analysis

Proportions of women who were fully, partly, and never screened were reported for the ID and reference groups. They were also reported separately for the 5 diagnostic groups, the group of women identified through institutions, and further for ID severity among women with an ID diagnosis.

Relative estimates comparing the odds of being never screened among women with ID compared with women in the reference group were computed using logistic regression analyses. We applied 2 different models: one age adjusted and the other further adjusted for region of Denmark, country of origin, and physical and psychiatric comorbidities. The analyses were conducted for the entire cohort of women with ID. They were further computed separately for the 5 diagnostic groups and the group of women identified through institutions, so every woman only appeared once in each model, even though she could be registered in more than 1 group. Last, among women with an ID diagnosis, a model with severity of ID as an independent variable was conducted with women in the reference group as comparison group.

We conducted several sensitivity analyses. First, we computed analyses only including women who were 50 years of age at study entry, since we had complete screening history on this group, as they had not been involved in previous local screening programs. Second, we conducted analyses leaving out the sixth screening round, since this included the period of the COVID-19 pandemic, where lockdowns in the community could interfere with screening participation, even though the screening program proceeded. Third, we restricted the analyses to women who were invited at least once to make sure that low participation was not driven by lack of invitations. Fourth, we computed analyses excluding those who were solely identified through institutions, as they were not registered with a diagnosis leading to ID. Additionally, we investigated whether the number of completed screenings differed between partly screened women with and without ID.

Data were analyzed from December 1, 2021, to June 31, 2022. Analyses were performed using Stata, version 17.0 (StataCorp LLC). We used 95% CIs to evaluate statistical significance.

## Results

We included 5595 women with ID and 49 423 age-matched women in the reference group (eFigure 1 in Supplement 1); 2747 women with ID (49%) and 24 723 in the reference group (50%) were 50 years of age at study entry. Women with ID were more likely to have a low educational level, live without a partner, receive a disability pension, and have comorbidities than the reference women (Table 1).

**Table 1.** Baseline Characteristics Among Women With ID and an Age-Matched Reference Group

Characteristics	Cohort, No. (%)^a^	
	Women with ID (n = 5595)	Age-matched women in reference group (n = 49 423)
Age at study entry, y		
50	2747 (49)	24 723 (50)
51-59	1824 (33)	15 739 (32)
60-69	1024 (18)	8961 (18)
Birth cohort		
1940s	1603 (29)	13 974 (28)
1950s	2056 (37)	17 672 (36)
1960s	1936 (35)	17 777 (36)
Region of Denmark		
Capital	1465 (26)	14 978 (30)
Zealand	940 (17)	7820 (16)
Southern	1319 (24)	10 094 (20)
Central	1256 (22)	11 271 (23)
Northern	615 (11)	5260 (11)
Educational level^b^		
Short	3750 (67)	8755 (18)
Medium	944 (17)	24 591 (50)
Long	136 (2)	15 103 (31)
Unknown	765 (14)	974 (2)
Cohabitation status		
Cohabitating^c^	1313 (23)	35 755 (72)
Living alone	4275 (76)	13 538 (27)
Unknown	7 (0.1)	130 (0.3)
Work status		
Working	328 (6)	35 041 (71)
Disability pension	4939 (88)	5523 (11)
Age pension	123 (2)	3819 (8)
Other^d^	205 (4)	5040 (10)
Country of origin		
Denmark	5400 (97)	45 725 (93)
Immigrant or descendant from a Western country	80 (1)	1511 (3)
Immigrant or descendant from a non-Western country	115 (2)	2187 (4)
Physical comorbidity^e^		
0	4175 (75)	43 222 (87)
1-2	1215 (22)	5565 (11)
≥3	205 (4)	636 (1)
Psychiatric comorbidity^f^		
No	3841 (69)	46 592 (94)
Yes	1754 (31)	2831 (6)

Abbreviation: ID, intellectual disability.

^a^ Percentages have been rounded and may not total 100.

^b^ Short indicates mandatory school (7-9 years); medium, secondary school and vocational education (10-12 years); and long, short-, medium-, or long-term higher education (>12 years).

^c^ Defined as married or 2 people of the opposite sex over the age of 16 years and with a maximum age difference of 15 years living at the same address with no other adult in residence. This definition therefore excludes homosexual unmarried partners and partners with more than 15 years’ difference in age. These categories, however, account for very few couples.

^d^ For example, receiving cash benefits, unemployed, absence for sickness, student, or receiving other public transfer payments.

^e^ Defined using the Charlson Comorbidity Index (CCI), which includes 19 groups of conditions, each of them weighted according to its potential impact on mortality. The CCI score is calculated as the sum of those weights (range, 1-6). A higher CCI score indicates increased severity of comorbid conditions. One of the groups of conditions in CCI is hemiplegia, which was omitted from the score among women with cerebral palsy due to overlap in diagnoses related to this diagnosis.

^f^ Indicates contact with a psychiatric hospital department for psychiatric diagnoses other than ID within the last 10 years (*International Statistical Classification of Diseases and Related Health Problems, Tenth Revision*, codes F00-F69 and F80-F99).

In all, 82% of the women in the ID group had a diagnosis of ID, 10% were diagnosed with Down syndrome, 4% had cerebral palsy with ID, 2% had a metabolic disorders diagnosis (primarily disorder of glycine metabolism or defects in posttranslational modification of lysosomal enzymes), 1% were diagnosed with congenital malformation and/or chromosomal disorders (primarily tuberous sclerosis), and 30% were found through institutions. These percentages do not sum to 100% because women may have been identified with more than 1 diagnosis or through more than 1 register. Only 540 (10%) were solely included through institutions.

### Breast Cancer Screening

**Figure 1.**
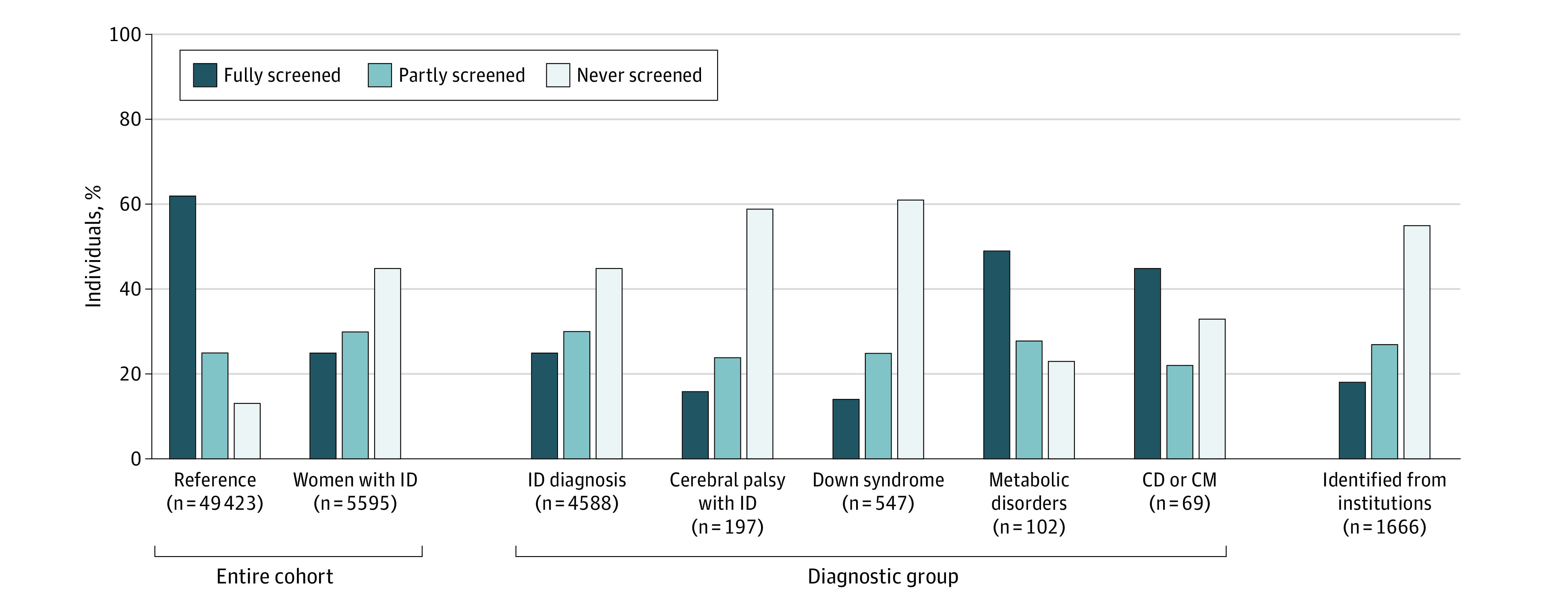
Screening Participation Overall and by Diagnostic Groups and Identification Through Institutions Participation during 6 invitation rounds in the Danish national breast cancer screening program (2007-2021) is shown among 5595 women with intellectual disability (ID) and 49 423 age-matched women in a reference group born between 1941 and 1967. Each woman in the ID group can appear in several diagnostic groups as well as institution identification; thus, the numbers sum to more than 5595 women. The women only appear once within each group. Fully screened women are those who were invited at least to their allocated potential screening rounds and participated in all of them. Partly screened women are those who were invited to fewer screening rounds than they should have been or participated in fewer rounds than they were invited to. Never screened women are those who were not invited to any screening rounds or did not participate in any of the rounds they were invited to. CD indicates chromosomal disorder; CM, congenital malformation.

In all, 1425 (25%) of women with ID were fully screened according to guidelines for the Danish breast cancer screening program, compared with 30 480 (62%) in the reference group (Figure 1). Among women with ID, 2498 (45%) were never screened (2155 [39%] did not attend any of the screenings they were invited to, and 343 [6%] were never invited). For women in the reference group, 6573 (13%) were never screened (5743 [11%] did not attend and 830 [2%] were not invited). Corresponding relative estimates show that women with ID had nearly 5 times higher odds for never being screened compared to reference women (odds ratio [OR], 4.90 [95% CI, 4.60-5.22]) (Table 2).

**Table 2.** Odds for Having Never Been Screened Among Women With ID Compared With an Age-Matched Reference Group

Group	No. never screened/total No. (%)	OR (95% CI)	
		Adjusted for age	Adjusted for age and categorical variables^a^
Reference group	6573/49 423 (13)	1 [Reference]	1 [Reference]
Women with ID	2498/5595 (45)	5.34 (5.04-5.67)	4.90 (4.60-5.22)
Diagnostic groups^b^			
ID diagnosis	2086/4588 (45)	5.52 (5.17-5.89)	5.16 (4.81-5.54)
Cerebral palsy and ID	117/197 (59)	11.07 (8.04-15.25)	11.31 (7.99-16.01)
Down syndrome	331/547 (61)	11.44 (9.42-13.89)	10.98 (8.95-13.47)
Metabolic disorders	23/102 (23)	1.95 (1.17-3.26)	1.45 (0.81-2.61)
Congenital malformations and/or chromosomal disorders	23/69 (33)	3.57 (2.05-6.22)	3.72 (2.10-6.57)
Identified from institutions (unknown diagnosis)	918/1666 (55)	8.58 (7.70-9.57)	7.97 (7.12-8.93)

Abbreviations: ID, intellectual disability; OR, odds ratio.

^a^ Categorical variables include region of Denmark (Northern, Central, Southern, Capital, or Zealand), country of origin (Danish, immigrant or descendant from a Western country, or immigrant or descendant from a non-Western country), physical comorbidity (Charlson Comorbidity Index 0, 1-2, or ≥3), and psychiatric comorbidity (yes or no).

^b^ The women with ID can appear in several diagnostic groups and also in the group identified through institutions, as they could be recorded with more than 1 of the inclusion diagnoses in the registers or identified both through an institution and also through 1 or more of the inclusion diagnoses. Thus, the numbers sum to more than 5595 women. Each diagnostic group and the group of women identified through institutions were analyzed in separate models. In each model, the women only appear once.

Screening participation varied across diagnostic groups. It was lowest among women with Down syndrome, among whom 78 (14%) were fully screened, followed by women with cerebral palsy including ID, among whom 32 (16%) were fully screened (Figure 1). Results from adjusted regression models showed that women with these diagnoses had 11 times higher odds for never being screened compared with women in the reference group (OR for cerebral palsy and ID, 11.31 [95% CI, 7.99-16.01]; OR for Down syndrome, 10.98 [95% CI, 8.95-13.47) (Table 2).

**Figure 2.**
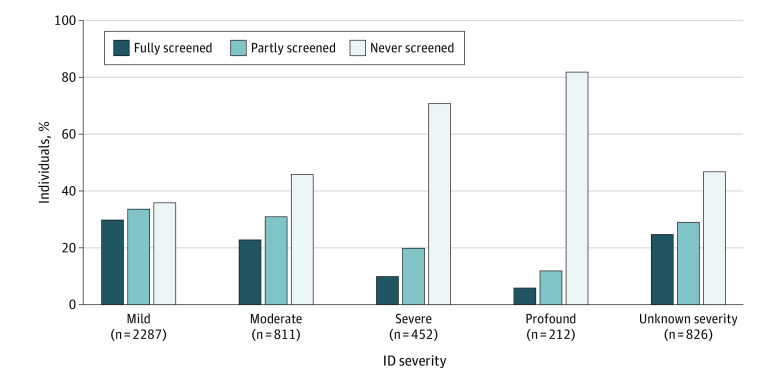
Screening Participation Among Women With an Intellectual Disability (ID) Diagnosis by ID Severity Participation during 6 invitation rounds in the Danish national breast cancer screening program (2007-2021) among 4588 women with an ID diagnosis. Severity of ID has been categorized hierarchically, and women only appear in the group with highest level of ID severity recorded for them in registers. Fully screened women are those who were invited at least to their allocated potential screening rounds and participated in all of them. Partly screened women are those who were invited to fewer screening rounds than they should have been or participated in fewer rounds than they were invited to. Never screened women are those who were not invited to any screening rounds or did not participate in any of the rounds they were invited to.

Among women with an ID diagnosis, the proportion who were never screened increased with severity of ID diagnosis, from 834 of 2287 (36%) among women with mild ID to 173 of 212 (82%) among women with profound ID (Figure 2). This pattern was also observed in the adjusted regression models, going from 3 times higher odds for never being screened among women with mild ID (OR, 3.36 [95% CI, 3.05-3.70]) to 30 times higher odds among women with profound ID (OR, 31.28 [95% CI, 21.98-44.52]) compared with the reference group (Table 3).

**Table 3.** Odds for Having Never Been Screened Among Women With an ID Diagnosis and an Age-Matched Reference Group Divided by ID Severity

ID severity^a^	No. never screened/total No. (%)	OR (95% CI)	
		Adjusted for age	Adjusted for age and categorical variables^b^
Mild	834/2287 (36)	3.70 (3.38-4.05)	3.36 (3.05-3.70)
Moderate	375/811 (46)	5.90 (5.12-6.81)	5.09 (4.39-5.91)
Severe	319/452 (71)	16.43 (13.38-20.18)	15.55 (12.61-19.18)
Profound	173/212 (82)	30.25 (21.32-42.93)	31.28 (21.98-44.52)
Unknown severity	385/826 (47)	5.88 (5.11-6.77)	5.80 (5.03-6.69)

Abbreviations: ID, intellectual disability; OR, odds ratio.

^a^ Categorized hierarchically. Women only appear in the group with highest level of ID severity with which they have been recorded in registers.

^b^ Categorical variables include region of Denmark (Northern, Central, Southern, Capital, or Zealand), country of origin (Danish, immigrant or descendant from a Western country, or immigrant or descendant from a non-Western country), physical comorbidity (Charlson Comorbidity Index 0, 1-2, or ≥3), and psychiatric comorbidity (yes or no).

### Sensitivity Analysis

When we only included women 50 years of age at study entry (with complete screening history), participation proportions increased slightly for women with ID as well as women in the reference group (eFigure 2 in Supplement 1). Relative estimates were similar to those in the complete cohort (eTable 2 in Supplement 1).

Leaving out the sixth screening round (during COVID-19) did not affect the results noticeably (eFigure 3 in Supplement 1). This was also the case when we only included women who were invited at least once (eFigure 4 in Supplement 1) and when we excluded those solely identified through institutions (eFigure 5 and eTable 2 in Supplement 1).

Among partly screened women who were eligible for at least 5 screenings rounds, there was a pattern toward lower participation among women with ID compared with the reference group. Most partly screened women without ID were screened 4 or 5 times, whereas the number of completed screenings varied more widely among partly screened women with ID (eTable 3 in Supplement 1).

## Discussion

In this dynamic population-based cohort study, we observed markedly lower participation in the Danish national breast cancer screening program among women with ID compared with women without ID. In addition, we found a clear stepwise decrease in participation with increased severity of ID.

Similar to our results, a few previous studies have found considerably lower participation in breast cancer screening among women with ID compared with women without ID. The proportions of participation differ widely across studies (4%-64%),^8,9,10,11,12,13,14,15^ and comparison of results are challenging. First, the studies were conducted in Taiwan,^11^ Australia,^9^ France,^12^ the US,^8,13^ Canada,^14^ and South Korea,^10^ where the health care systems, screening programs, and access to cancer screening differ widely. Second, various definitions of screening participation were applied. Third, the results were based on data from different sources—that is, some data were self-reported,^12,13^ some were obtained from medical records,^15^ and some were register-based.^8,9,10,11,14^ Fourth, in most of the studies, selected populations were included, for example those with health insurance,^8^ those receiving disability services^9^ or welfare benefits,^10^ or those residing at institutions.^12^

We found that the disparity in participation was mainly driven by women with ID who were never screened, whereas the proportion of women who were partly screened was relatively similar between women with and without ID. This could indicate that women with ID are already more likely not to initiate participation in the screening program. Looking more closely into the partly screened groups, however, did show that screening participation also was lower among partly screened women with ID than partly screened women without ID.

A few studies have suggested different reasons for this markedly lower breast cancer screening participation among women with ID, none from a Danish setting. A recently published systematic review^35,36^ shows that barriers for screening participation in this group are perceptions of fear, distress, and embarrassment; unpreparedness for screening; negative interactions with health care professionals; a lack of knowledge about cancer screening; mobility issues; and a lack of ability to provide consent and communicate verbally.

Breast cancer screening programs are not designed to accommodate the challenges associated with having ID. Mammography is a demanding examination, and it requires that the woman understand information and instructions given. The examination can be unpleasant, and the woman must be able to cooperate and place her body and breasts in several positions.^35^ In a recently published review,^37^ the authors suggest that breast cancer screening should not be recommended for women with Down syndrome. Instead, they suggest annual clinical monitoring with palpation by a health care professional, with the option to perform ultrasound or magnetic resonance imaging examination if needed.^37^ However, no evidence exists on the effect of these examinations as a screening strategy. In Denmark, it is possible to complete the screening mammography in a wheelchair, and women with special needs can be given extra time for the examination. Further, individuals with disability who are unable to use public transport are eligible for door-to-door transportation services, and if the disability prevents them from traveling alone, they can be granted accompaniment. However, no guidelines or accommodated approaches for breast cancer screening among women with ID are available. This is warranted to guide the women as well as family caregivers and health care professionals on how to support the women in the most optimal way, sparing them psychological distress, but also securing early detection of breast cancer.^38^

### Strengths and Limitations

To our knowledge, this study is the first to investigate participation in breast cancer screening among women with ID in Denmark, where the screening program is tax-financed and thus access to screening is independent of income and health insurance. Furthermore, because the study was population-based, the results may be generalized to settings that are comparable to the Danish health care system.

This study has some limitations. It was a challenge to identify all women with ID, since they are not systematically registered. We used different high-quality data sources, including both hospital contacts and residence at institutions for persons with ID, and we are confident that we identified most women with ID, especially those with severe and profound disability. However, women with mild ID may be slightly underrepresented, since they do not reside at institutions, and a few might not have had any hospital contacts.

Some women with ID have diagnoses that lead to markedly increased mortality rates (L.C.T., T.H.L., T.A.H., et al; unpublished data; June 2022). Thus, women in this study are a select group who have survived at least to 50 years of age. This is especially an issue in the group of women with metabolic disorders, among whom it could be suspected that those who survived beyond 50 years of age have no or very mild ID. This is supported by the results showing only slightly lower screening participation in this group compared with the reference group.

## Conclusions

In this population-based cohort study, women with ID were markedly less likely to participate in breast cancer screening compared with women without ID. Screening participation decreased with increasing severity of ID. These findings suggest a need for studies focusing on barriers and facilitators for participating in breast cancer screening in a Danish setting among women with ID. Such knowledge could facilitate tailored guidelines and approaches for breast cancer screening in this group of women.

## Notes

Supplement 1. **eTable 1.** Diagnoses Used to Identify Persons With Intellectual Disability (ID) **eFigure 1.** Flowchart of Inclusion **eFigure 2.** Participation During 6 Invitation Rounds in the Danish National Breast Cancer Screening Program (2007-2021) Among Women With Complete Screening History **eFigure 3.** Participation During 6 Invitation Rounds in the Danish National Breast Cancer Screening Program (2007-2018) Leaving Out the Sixth Screening Round (During the COVID-19 Pandemic) **eFigure 4.** Participation During 6 Invitation Rounds in the Danish National Breast Cancer Screening Program (2007-2021) Among Women Who Were Invited to Screening at Least Once **eFigure 5.** Participation During 6 Invitation Rounds in the Danish National Breast Cancer Screening Program (2007-2021) Excluding Women Who Were Solely Identified Through Institutions **eTable 2.** Odds for Having Never Been Screened Among Women with Intellectual Disability (ID) and Age-Matched Reference Women: Primary Analyses and 4 Sensitivity Analyses **eTable 3.** Number of Completed Screenings Among Partly Screened Women Who Were Eligible for at Least 5 Screenings Rounds

Supplement 2.
Data Sharing Statement
