# Supplementary material for: Breast Cancer Screening Among Women With Intellectual Disability in Denmark
Source: JAMA Netw Open. 2023 Jan 3;6(1):e2248980. doi: 10.1001/jamanetworkopen.2022.48980 (PMC9856850; doi:10.1001/jamanetworkopen.2022.48980)
Supplement: Supplement 1. — eTable 1. Diagnoses Used to Identify Persons With Intellectual Disability (ID) eFigure 1. Flowchart of Inclusion eFigure 2. Participation During 6 Invitation Rounds in the Danish National Breast Cancer Screening Program (2007-2021) Among Women With Complete Screening History eFigure 3. Participation During 6 Invitation Rounds in the Danish National Breast Cancer Screening Program (2007-2018) Leaving Out the Sixth Screening Round (During the COVID-19 Pandemic) eFigure 4. Participation During 6 Invitation Rounds in the Danish National Breast Cancer Screening Program (2007-2021) Among Women Who Were Invited to Screening at Least Once eFigure 5. Participation During 6 Invitation Rounds in the Danish National Breast Cancer Screening Program (2007-2021) Excluding Women Who Were Solely Identified Through Institutions eTable 2. Odds for Having Never Been Screened Among Women with Intellectual Disability (ID) and Age-Matched Reference Women: Primary Analyses and 4 Sensitivity Analyses eTable 3. Number of Completed Screenings Among Partly Screened Women Who Were Eligible for at Least 5 Screenings Rounds [file jamanetwopen-e2248980-s001.pdf]

Supplementary Online Content

Horsbøl TA, Michelsen SI, Lassen TH, et al. Breast cancer screening among women with intellectual disability in Denmark. *JAMA Netw Open*. 2023;6(1):e2248980.
doi:10.1001/jamanetworkopen.2022.48980

eTable 1. Diagnoses Used to Identify Persons With Intellectual Disability (ID)
eFigure 1. Flowchart of Inclusion
eFigure 2. Participation During 6 Invitation Rounds in the Danish National Breast Cancer Screening Program (2007-2021) Among Women With Complete Screening History
eFigure 3. Participation During 6 Invitation Rounds in the Danish National Breast Cancer Screening Program (2007-2018) Leaving Out the Sixth Screening Round (During the COVID-19 Pandemic)
eFigure 4. Participation During 6 Invitation Rounds in the Danish National Breast Cancer Screening Program (2007-2021) Among Women Who Were Invited to Screening at Least Once
eFigure 5. Participation During 6 Invitation Rounds in the Danish National Breast Cancer Screening Program (2007-2021) Excluding Women Who Were Solely Identified Through Institutions
eTable 2. Odds for Having Never Been Screened Among Women with Intellectual Disability (ID) and Age-Matched Reference Women: Primary Analyses and 4 Sensitivity Analyses
eTable 3. Number of Completed Screenings Among Partly Screened Women Who Were Eligible for at Least 5 Screenings Rounds

This supplementary material has been provided by the authors to give readers additional information about their work.

© 2023 Horsbøl TA et al. *JAMA Network Open*.

eTable 1. Diagnoses Used to Identify Persons With Intellectual Disability (ID)

**Diagnosis**	**ICD-8**	**ICD-10**
Mild ID	310-311	F70
Moderate ID	312	F71
Severe ID	313	F72
Profound ID	314	F73
Other ID and ID without further specification	315	F78, F79
Cerebral palsy and ID	-	G80[a]
Down's syndrome	759.3	Q90
Metabolic disorders likely to result in ID	271.2	E72.0E, E72.5A, E72.8E, E74.2B, E74.4B, E74.4C, E75.0, E75.1, E75.2D, E75.2E, E75.2G, E75.2H, E75.3, E75.4, E75.5A, E75.5B, E77, E79.1
Congenital malformation and chromosomal disorders likely to result in ID	759.6	Q85.1, Q93.5C, Q93.8A, Q99

[a] Persons with cerebral palsy are not identified by ICD-10 codes but through child neurologist medical record review.

© 2023 Horsbøl TA et al. *JAMA Network Open*.

eFigure 1. Flowchart of Inclusion

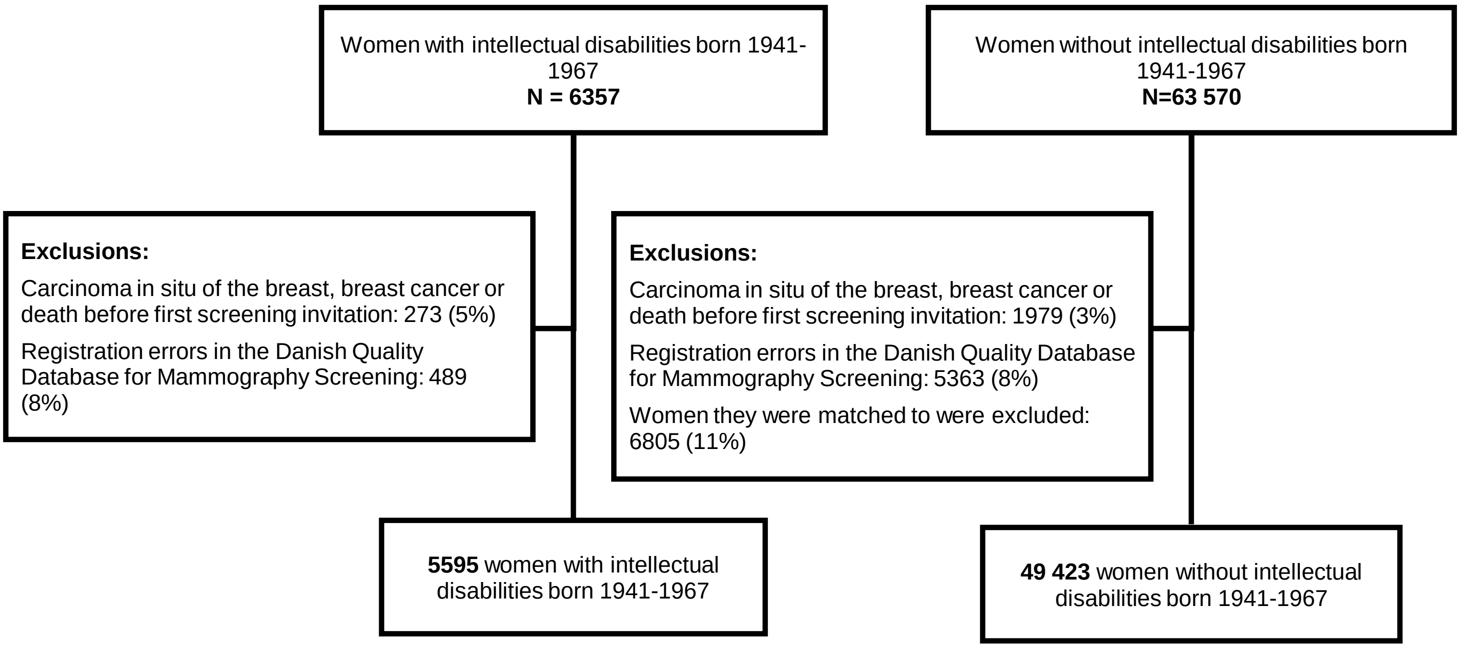

© 2023 Horsbøl TA et al. *JAMA Network Open*.

eFigure 2. Participation During 6 Invitation Rounds in the Danish National Breast Cancer Screening Program (2007-2021) Among Women With Complete Screening History

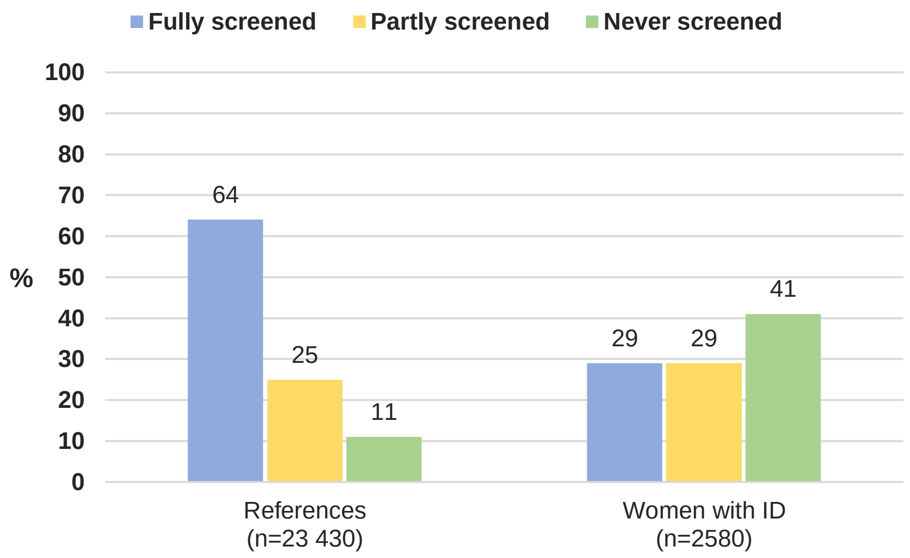

Includes 2580 Women with Intellectual Disability (ID) and 23 430 Age-Matched Reference Women Born 1957-1967.

© 2023 Horsbøl TA et al. *JAMA Network Open*.

eFigure 3. Participation During 6 Invitation Rounds in the Danish National Breast Cancer Screening Program (2007-2018) Leaving Out the Sixth Screening Round (During the COVID-19 Pandemic)

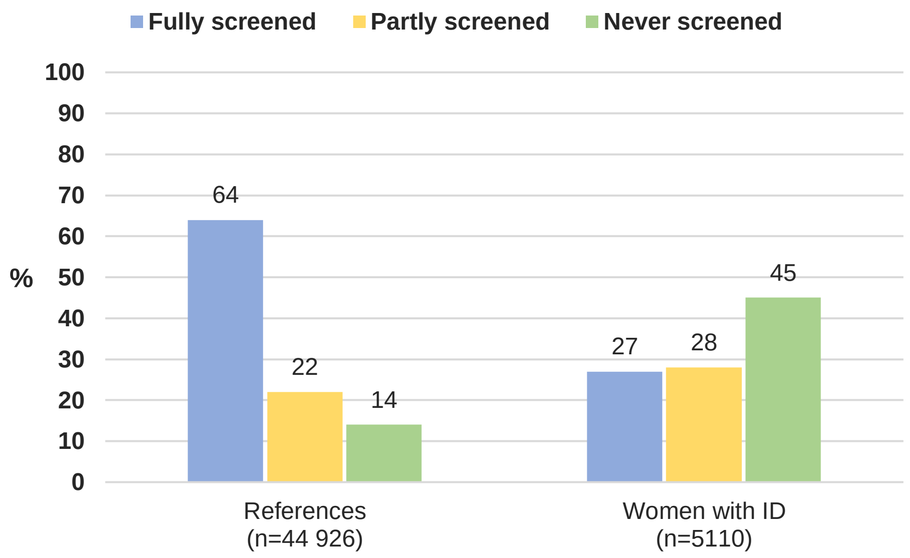

Includes 5110 Women with Intellectual Disability (ID) and 44 926 Age-Matched Reference Women born 1941-1965.

© 2023 Horsbøl TA et al. *JAMA Network Open*.

eFigure 4. Participation During 6 Invitation Rounds in the Danish National Breast Cancer Screening Program (2007-2021) Among Women Who Were Invited to Screening at Least Once

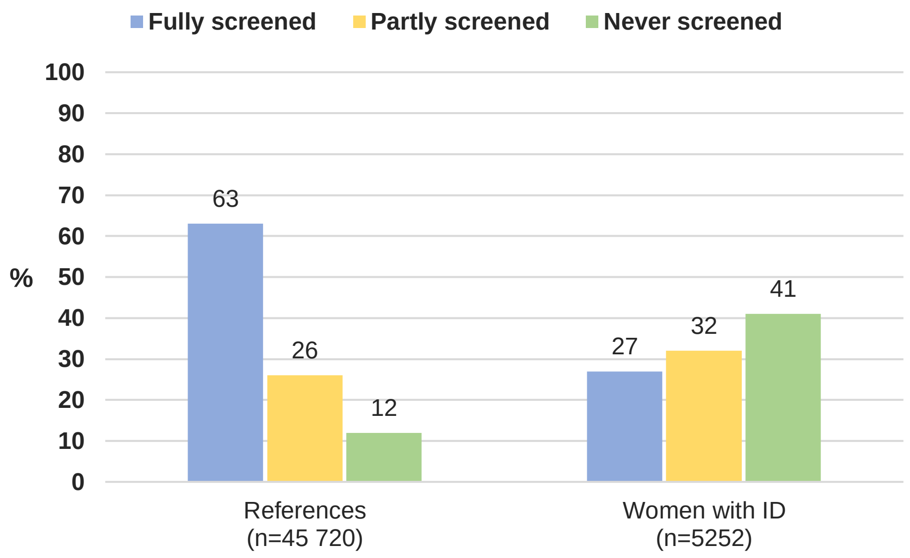

Includes 5252 Women with Intellectual Disability (ID) and 45 720 Age-Matched Reference Women born 1941-1967.

© 2023 Horsbøl TA et al. *JAMA Network Open*.

eFigure 5. Participation During 6 Invitation Rounds in the Danish National Breast Cancer Screening Program (2007-2021) Excluding Women Who Were Solely Identified Through Institutions

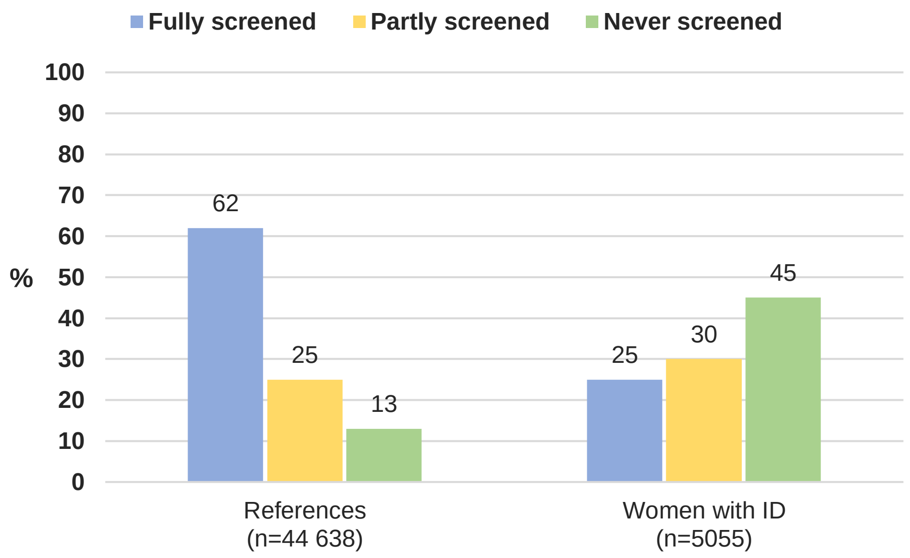

Includes 5055 Women with Intellectual Disability (ID) and 44 638 Age-Matched Reference Women born 1941-1967.

© 2023 Horsbøl TA et al. *JAMA Network Open*.

eTable 2. Odds for Having Never Been Screened Among Women with Intellectual Disability (ID) and Age-Matched Reference Women: Primary Analyses and 4 Sensitivity Analyses

	**Never screened No. / total No. (%)**	**OR[a] (95% CI)**	**OR[b] (95% CI)**
The primary analysis of women born 1941-1967 (same as Table 2 in the paper)			
References	6573/49 423 (13)	1	1
Women with ID	2498/5595 (45)	5.34 (5.04 – 5.67)	4.90 (4.60 – 5.22)
Women born 1957-1967 (with complete screening history)			
References	2625/23 430 (11)	1	1
Women with ID	1063/2580 (41)	5.55 (5.08 – 6.07)	5.23 (4.75 – 5.75)
Women born 1941-1965, leaving out the 6th screening round (during the Covid 19 pandemic)			
References	6203/44 926 (14)	1	1
Women with ID	2320/5110 (45)	5.27 (4.96 – 5.61)	4.80 (4.49 – 5.13)
Women born 1941-1967, who were invited to screening at least once			
References	5365/45 720 (12)	1	1
Women with ID	2155/5252 (41)	5.25 (4.93 – 5.58)	4.84 (4.53 – 5.17)
Women born 1941-1967, excluding those who were solely identified through institutions			
References	5910/44 638 (13)	1	1
Women with ID	2272/5055 (45)	5.44 (5.11 – 5.79)	5.04 (4.72 – 5.39)

[a] Adjusted for age (continuous variable).
[b] Adjusted for age (continuous variable), region (Northern, Central, Southern, Capital, Zealand), country of origin (Danish, immigrant/descendant from a western country, immigrant/descendant from a non-western country), physical comorbidity (Charlson Comorbidity Index: 0, 1-2, ≥3), and psychiatric comorbidity (yes, no).

© 2023 Horsbøl TA et al. *JAMA Network Open*.

eTable 3. Number of Completed Screenings Among Partly Screened Women Who Were Eligible for at Least 5 Screening Rounds

		Women with ID		Age-matched reference group	
**Screening status**	**Completed screenings**	**No.**	**(%)**	**No.**	**(%)**
**Never**		735	(41)	1944	(11)
**Partly**	**1**	158	(9)	628	(4)
	**2**	122	(7)	613	(3)
	**3**	113	(6)	795	(4)
	**4**	149	(8)	1540	(9)
	**5**	116	(7)	1916	(11)
**Fully**		382	(22)	10366	(58)
**In all**		1775		17802	

Includes Women Born 1949-1959, Alive and without Carcinoma in Situ in the Breast or Breast Cancer During Follow-up.

© 2023 Horsbøl TA et al. *JAMA Network Open*.
