# Supplementary material for: Breast Cancer Screening Among Women With Intellectual Disability in Denmark
Source: JAMA Netw Open. 2023 Jan 3;6(1):e2248980. doi: 10.1001/jamanetworkopen.2022.48980 (PMC9856850; doi:10.1001/jamanetworkopen.2022.48980)
Supplement: Supplement 2. — Data Sharing Statement [file jamanetwopen-e2248980-s002.pdf]

Data Sharing Statement

Horsbøl. Breast Cancer Screening Among Women With Intellectual Disability in Denmark. *JAMA Netw Open*. Published January 3, 2023. doi:10.1001/jamanetworkopen.2022.48980

Data
Data available: No

Additional Information
Explanation for why data not available: All data related to the study are stored at a secured server at Statistics Denmark and are not available to other researchers.
